# Supplementary material for: Preadmission kidney function and risk of acute kidney injury in patients hospitalized with acute pyelonephritis: A Danish population-based cohort study
Source: PLoS One. 2021 Mar 3;16(3):e0247687. doi: 10.1371/journal.pone.0247687 (PMC7929569; doi:10.1371/journal.pone.0247687)
Supplement: S1 Table — NPU codes and analysis codes for identifying creatinine measurements in the laboratory databases. (DOCX) [file pone.0247687.s001.docx]

S1 Table

| **ICD-10 codes used for inclusion** | |
| --- | --- |
| Pyelonephritis: | DN109 (until 2011), DN109C, DN12, DN129, DN209A, DO230, DO862B |
| **ICD-10 and procedure codes used for exclusion** | |
| Chronic pyelonephritis: | DN11* |
| Chronic dialysis: | BJFD2* |
| Muscular dystrophy, paraplegia and tetraplegia: | DG114, DG12*, DG710*, DG711*, DG712*, DG80*, DG81*, DG82* |
| **ICD-10, ICD-8 and ATC codes used for identifying covariates** | |
| Diabetes: | ICD-8: “249.00”, ”249.06”, ”249.07”, ”249.09”, ”250.00”, ”250.06”, ”250.07”, ”250.09”  ICD-10: DE10*, DE11*, DE12*, DE13*, DE14*, DG63.2, DH360, DN083, DO24* (except DO244) |
| Diabetes ATC: | A10A*, A10B* |
| Hypertension: | ICD-8: 400*, 401*, 402*, 403*, 404*  ICD-10: DI10*, DI11*, DI12*, DI13*, DI14*, DI15* |
| Heart failure: | ICD-8: 42709, 42710, 42711, 42719, 42899, 78249  ICD-10: DI500*, DI501*, DI502*, DI503*, DI508*, DI509*, DI110*, DI130*, DI132*, DI420*, DI426*, DI427*, DI428*, DI429* |
| Malformation of the urinary tract: | ICD-8: 598*, 753*  ICD-10: DQ62*, DQ64* |
| Obstructive nephropathy | ICD-10: DN13* (Five years prior to pyelonephritis admission) |
| Nephrolithiasis | ICD-10: DN20* (Five years prior to pyelonephritis admission) |
| **Codes for identifying serum creatinine** | |
| NPU codes: | NPU18016, NPU01807, NPU04998, ASS00354, ASS00355, AAB00327 |
| Analysis codes: | 716, 1807, 4998, 18016, 110266, 1511235, 1610154, 1611807, 1710301, 1711807, 1811807, 1817156 |
| **ICD-8, ICD-10, and procedure codes used for multiple imputation** | |
| Acute dialyses: | BJFD0* |
| Catheter a demeure: | BJAA1, BJAZ00, BJAZ10, BJAZ20, BJAZ30, DZ978A |
| **Charlson comorbidity score:** | |
| Myocardial infarction: | ICD-8: 410  ICD-10: I21; I22; I23 |
| Congestive heart failure: | ICD-8: 427.09; 427.10; 427.11; 427.19; 428.99; 782.49  ICD-10: I50; I11; I13; I13.2 |
| Peripheral vascular disease: | ICD-8: 440; 441; 442; 443; 444; 445  ICD-10: I70; I71; I72; I73; I74; I77 |
| Cerebrovascular disease: | ICD-8: 430-438  ICD-10: I60-I69; G45; G46 |
| Mild liver disease: | ICD-8: 571; 573.01; 573.04  ICD-10: B18; K70.0-K70.3; K70.9; K71; K73; K74; K76.0 |
| Diabetes: | ICD-8: 249.00; 249.06; 249.07; 249.09; 250.00; 250.06; 250.07; 250.09  ICD-10: E10.0; E10.1; E10.9; E11.0; E11.1; E11.9 |
| Hemiplegia: | ICD-8: 344  ICD-10: G81; G82 |
| Moderate to severe renal disease: | ICD-8: 403; 404; 580-583; 584; 590.09; 593.19; 753.10-753.19; 792  ICD-10: I12; I13; N00-N05; N07; N11; N14; N17-N19; Q61 |
| Diabetes with end organ disease: | ICD-8: 249.01-249.05; 249.08; 250.01-250.05; 250.08  ICD-10: E10.2-E10.8; E11.2-E11.8 |
| Any tumor: | ICD-8: 140-194  ICD-10: C00-C75 |
| Leukemia: | ICD-8: 204-207  ICD-10: C91-C95 |
| Lymphoma: | ICD-8: 200-203; 275.59  ICD-10: C81-C85; C88; C90; C96 |
| Moderate to severe liver disease: | ICD-8: 070.00; 070.02; 070.04; 070.06; 070.08; 573.00; 456.00-456.09  ICD-10: B15.0; B16.0; B16.2; B19.0; K70.4; K72; K76.6; I85 |
| Metastatic solid tumor: | ICD-8: 195-198; 199  ICD-10: C76-C80 |
| AIDS/HIV: | ICD-8: 079.83  ICD-10: B21-B24 |
