# Supplementary material for: Preadmission kidney function and risk of acute kidney injury in patients hospitalized with acute pyelonephritis: A Danish population-based cohort study
Source: PLoS One. 2021 Mar 3;16(3):e0247687. doi: 10.1371/journal.pone.0247687 (PMC7929569; doi:10.1371/journal.pone.0247687)
Supplement: S2 Table — (DOCX) [file pone.0247687.s002.docx]

S2 Table

| **Preadmission eGFR** | **Sex/age adjusted** | **95% CI** | **Fully adjusted** | **95% CI** |
| --- | --- | --- | --- | --- |
| **≥90** | 1.00 *(reference)* | - | 1.00 *(reference)* | - |
| **60-89** | 1.01 | 0.91 ; 1.12 | 1.02 | 0.92 ; 1.13 |
| **45-59** | 1.50 | 1.29 ; 1.74 | 1.48 | 1.27 ; 1.72 |
| **30-44** | 2.21 | 1.87 ; 2.62 | 2.15 | 1.80 ; 2.55 |
| **<30** | 2.69 | 2.20 ; 3.30 | 2.61 | 2.12 ; 3.20 |
