# Supplementary material for: Preadmission kidney function and risk of acute kidney injury in patients hospitalized with acute pyelonephritis: A Danish population-based cohort study
Source: PLoS One. 2021 Mar 3;16(3):e0247687. doi: 10.1371/journal.pone.0247687 (PMC7929569; doi:10.1371/journal.pone.0247687)
Supplement: S3 Table — (DOCX) [file pone.0247687.s003.docx]

S3 Table

| **Preadmission eGFR** | **Sex/age adjusted** | **95% CI** | **Fully adjusted** | **95% CI** |
| --- | --- | --- | --- | --- |
| **≥90** | 1.00 *(reference)* | - | 1.00 *(reference)* | - |
| **60-89** | 0.94 | 0.81 ; 1.10 | 0.95 | 0.81 ; 1.11 |
| **45-59** | 1.34 | 1.10 ; 1.64 | 1.29 | 1.06 ; 1.58 |
| **30-44** | 1.77 | 1.43 ; 2.20 | 1.65 | 1.33 ; 2.05 |
| **<30** | 2.10 | 1.65 ; 2.68 | 1.97 | 1.54 ; 2.51 |
