# Supplementary material for: Preadmission kidney function and risk of acute kidney injury in patients hospitalized with acute pyelonephritis: A Danish population-based cohort study
Source: PLoS One. 2021 Mar 3;16(3):e0247687. doi: 10.1371/journal.pone.0247687 (PMC7929569; doi:10.1371/journal.pone.0247687)
Supplement: S4 Table — (DOCX) [file pone.0247687.s004.docx]

S4 Table

|  | **Females** | | **Males** | | **Test for statistical interaction** |
| --- | --- | --- | --- | --- | --- |
| **Preadmission eGFR** | Sex/age adjusted OR (95% CI) | Fully adjusted OR (95% CI) | Sex/age adjusted OR (95% CI) | Fully adjusted OR (95% CI) | P-value |
|  |  |  |  |  |  |
| **≥90** | 1.00  *(reference)* | 1.00  *(reference)* | 1.00  *(reference)* | 1.00  *(reference)* | 0.0716 |
| **60-89** | 0.92  (0.76 – 1.11) | 0.92  (0.76 – 1.11) | 0.99  (0.77 – 1.26) | 1.00  (0.78 – 1.28) |  |
| **45-59** | 1.15  (0.90 – 1.48) | 1.08  (0.84 – 1.39) | 1.79  (1.33 – 2.42) | 1.77  (1.31 – 2.40) |  |
| **30-44** | 1.65  (1.25-2.18) | 1.49  (1.12 – 1.97) | 2.40  (1.75 – 3.31) | 2.32  (1.68 – 3.20) |  |
| **<30** | 2.60  (1.89-3.59) | 2.32  (1.68 – 3.20) | 2.21  (1.55 – 3.17) | 2.15  (1.50 – 3.09) |  |
