# Supplementary material for: Preadmission kidney function and risk of acute kidney injury in patients hospitalized with acute pyelonephritis: A Danish population-based cohort study
Source: PLoS One. 2021 Mar 3;16(3):e0247687. doi: 10.1371/journal.pone.0247687 (PMC7929569; doi:10.1371/journal.pone.0247687)
Supplement: S5 Table — (DOCX) [file pone.0247687.s005.docx]

S5 Table

| **Preadmission eGFR** | **Fully adjusted** | **95% CI** |
| --- | --- | --- |
| **≥90** | 1.00 *(reference)* | - |
| **60-89** | 0.93 | 0.80 ; 1.08 |
| **45-59** | 1.23 | 1.01 ; 1.49 |
| **30-44** | 1.62 | 1.31 ; 2.00 |
| **<30** | 1.95 | 1.53 ; 2.48 |
