# Supplementary material for: Preadmission kidney function and risk of acute kidney injury in patients hospitalized with acute pyelonephritis: A Danish population-based cohort study
Source: PLoS One. 2021 Mar 3;16(3):e0247687. doi: 10.1371/journal.pone.0247687 (PMC7929569; doi:10.1371/journal.pone.0247687)
Supplement: S1 File — (DOCX) [file pone.0247687.s006.docx]

# **Main regression analyses**

## **Sex/age adjusted**

logistic AKIvar_senest ib90.groupeGFR c.age50 gender

Logistic regression Number of obs = 8,760

LR chi2(6) = 560.11

Prob > chi2 = 0.0000

Log likelihood = -4553.8506 Pseudo R2 = 0.0579

----------------------------------------------------------------------------------

AKIvar_senest | Odds Ratio Std. Err. z P>|z| [95% Conf. Interval]

-----------------+----------------------------------------------------------------

groupeGFR |

eGFR < 30 | 2.355287 .2841803 7.10 0.000 1.859263 2.983642

30 <= eGFR < 45 | 1.925861 .2043476 6.18 0.000 1.564251 2.371066

45 <= eGFR < 60 | 1.377087 .1341091 3.29 0.001 1.137801 1.666697

60 <= eGFR < 90 | .9472693 .0724108 -0.71 0.479 .8154667 1.100375

|

age50 | 1.021569 .0019714 11.06 0.000 1.017713 1.025441

gender | .8583056 .0466326 -2.81 0.005 .7716056 .9547475

_cons | .2280026 .0142577 -23.64 0.000 .2017027 .2577319

----------------------------------------------------------------------------------

## **Fully adjusted**

logistic AKIvar_senest ib90.groupeGFR gender c.age50 diabetes MUT hypertension HF

Logistic regression Number of obs = 8,760

LR chi2(10) = 626.57

Prob > chi2 = 0.0000

Log likelihood = -4520.6202 Pseudo R2 = 0.0648

----------------------------------------------------------------------------------

AKIvar_senest | Odds Ratio Std. Err. z P>|z| [95% Conf. Interval]

-----------------+----------------------------------------------------------------

groupeGFR |

eGFR < 30 | 2.194589 .2680632 6.43 0.000 1.727354 2.788209

30 <= eGFR < 45 | 1.784426 .1920369 5.38 0.000 1.445086 2.203451

45 <= eGFR < 60 | 1.32057 .1298434 2.83 0.005 1.089101 1.601234

60 <= eGFR < 90 | .9510603 .0730848 -0.65 0.514 .8180823 1.105654

|

gender | .8717332 .0477236 -2.51 0.012 .78304 .9704724

age50 | 1.019358 .0020275 9.64 0.000 1.015392 1.02334

diabetes | 1.492119 .0930477 6.42 0.000 1.320453 1.686102

MUT | .9047765 .1986139 -0.46 0.648 .5884209 1.391216

hypertension | 1.164781 .0674324 2.63 0.008 1.039838 1.304736

HF | 1.203493 .1064675 2.09 0.036 1.01191 1.431349

_cons | .2008086 .0130886 -24.63 0.000 .1767264 .2281724

----------------------------------------------------------------------------------

# **Regression analyses with multiple imputation**

## **Sex/age adjusted**

logistic AKIvar_senest ib90.groupeGFR c.age50 gender

Logistic regression Number of obs = 16,047

LR chi2(6) = 331.40

Prob > chi2 = 0.0000

Log likelihood = -9262.0371 Pseudo R2 = 0.0176

----------------------------------------------------------------------------------

AKIvar_senest | Odds Ratio Std. Err. z P>|z| [95% Conf. Interval]

-----------------+----------------------------------------------------------------

groupeGFR |

eGFR < 30 | 2.692987 .2783045 9.59 0.000 2.199214 3.297622

30 <= eGFR < 45 | 2.212916 .1925395 9.13 0.000 1.865968 2.624373

45 <= eGFR < 60 | 1.496202 .1146091 5.26 0.000 1.287621 1.73857

60 <= eGFR < 90 | 1.010049 .0537467 0.19 0.851 .9100147 1.12108

|

age50 | 1.003163 .0012556 2.52 0.012 1.000705 1.005627

gender | .9715028 .0409004 -0.69 0.492 .8945576 1.055066

_cons | .3349236 .014703 -24.92 0.000 .3073111 .365017

----------------------------------------------------------------------------------

## **Fully adjusted**

. logistic AKIvar_senest ib90.groupeGFR gender c.age50 diabetes MUT hypertension HF

Logistic regression Number of obs = 16,047

LR chi2(10) = 362.61

Prob > chi2 = 0.0000

Log likelihood = -9246.4305 Pseudo R2 = 0.0192

----------------------------------------------------------------------------------

AKIvar_senest | Odds Ratio Std. Err. z P>|z| [95% Conf. Interval]

-----------------+----------------------------------------------------------------

groupeGFR |

eGFR < 30 | 2.60627 .2731607 9.14 0.000 2.122295 3.200612

30 <= eGFR < 45 | 2.145197 .1893521 8.65 0.000 1.804402 2.550357

45 <= eGFR < 60 | 1.480681 .1145459 5.07 0.000 1.272367 1.723101

60 <= eGFR < 90 | 1.020693 .0545302 0.38 0.701 .9192209 1.133366

|

gender | .975708 .0412534 -0.58 0.561 .8981123 1.060008

age50 | 1.001822 .0013003 1.40 0.161 .9992769 1.004374

diabetes | 1.206747 .0643275 3.53 0.000 1.08703 1.339649

MUT | .6416018 .109674 -2.60 0.009 .4589476 .8969497

hypertension | 1.037967 .0504466 0.77 0.443 .9436569 1.141703

HF | 1.218568 .095208 2.53 0.011 1.04555 1.420218

_cons | .3221896 .0145381 -25.10 0.000 .294919 .3519817

# **Regression analyses within seven days**

## **Sex/age adjusted**

logistic AKIvar_senest ib90.groupeGFR c.age50 gender

Logistic regression Number of obs = 8,760

LR chi2(6) = 450.33

Prob > chi2 = 0.0000

Log likelihood = -4332.6584 Pseudo R2 = 0.0494

----------------------------------------------------------------------------------

AKIvar_senest | Odds Ratio Std. Err. z P>|z| [95% Conf. Interval]

-----------------+----------------------------------------------------------------

groupeGFR |

eGFR < 30 | 2.104295 .2605422 6.01 0.000 1.65088 2.68224

30 <= eGFR < 45 | 1.774071 .1943771 5.23 0.000 1.431227 2.199041

45 <= eGFR < 60 | 1.343313 .1355031 2.93 0.003 1.102337 1.636967

60 <= eGFR < 90 | .9435223 .074937 -0.73 0.464 .8075093 1.102445

|

age50 | 1.02058 .0020409 10.19 0.000 1.016588 1.024588

gender | .8658505 .0486022 -2.57 0.010 .7756451 .9665467

_cons | .2017339 .0130751 -24.70 0.000 .1776682 .2290594

----------------------------------------------------------------------------------

## **Fully adjusted**

logistic AKIvar_senest ib90.groupeGFR c.age50 gender diabetes MUT hypertension HF

Logistic regression Number of obs = 8,760

LR chi2(10) = 507.49

Prob > chi2 = 0.0000

Log likelihood = -4304.0768 Pseudo R2 = 0.0557

----------------------------------------------------------------------------------

AKIvar_senest | Odds Ratio Std. Err. z P>|z| [95% Conf. Interval]

-----------------+----------------------------------------------------------------

groupeGFR |

eGFR < 30 | 1.965819 .2462876 5.39 0.000 1.537803 2.512964

30 <= eGFR < 45 | 1.647845 .1830033 4.50 0.000 1.325517 2.048555

45 <= eGFR < 60 | 1.290524 .1313359 2.51 0.012 1.057157 1.575405

60 <= eGFR < 90 | .9469254 .0755564 -0.68 0.494 .8098365 1.107221

|

age50 | 1.018448 .0020988 8.87 0.000 1.014343 1.02257

gender | .8782641 .0496453 -2.30 0.022 .7861576 .9811618

diabetes | 1.463591 .0938722 5.94 0.000 1.2907 1.659642

MUT | .8783617 .2012897 -0.57 0.571 .5605416 1.376382

hypertension | 1.160831 .0694335 2.49 0.013 1.032418 1.305216

HF | 1.184151 .1071335 1.87 0.062 .9917369 1.413897

_cons | .1787447 .0120652 -25.51 0.000 .1565948 .2040277

# **Regression analyses stratified by gender**

## **Sex/age adjusted**

. bysort gender: logistic AKIvar_senest ib90.groupeGFR c.age50

----------------------------------------------------------------------------------------------------------------------

-> gender = 0

Logistic regression Number of obs = 2,988

LR chi2(5) = 118.92

Prob > chi2 = 0.0000

Log likelihood = -1747.3058 Pseudo R2 = 0.0329

----------------------------------------------------------------------------------

AKIvar_senest | Odds Ratio Std. Err. z P>|z| [95% Conf. Interval]

-----------------+----------------------------------------------------------------

groupeGFR |

eGFR < 30 | 2.212845 .4042857 4.35 0.000 1.54681 3.165666

30 <= eGFR < 45 | 2.40374 .3915295 5.38 0.000 1.74679 3.307761

45 <= eGFR < 60 | 1.789813 .2744124 3.80 0.000 1.325264 2.417203

60 <= eGFR < 90 | .9877775 .1227258 -0.10 0.921 .7742873 1.260132

|

age50 | 1.010387 .0036788 2.84 0.005 1.003203 1.017623

_cons | .2611723 .0245073 -14.31 0.000 .2172971 .3139065

----------------------------------------------------------------------------------

----------------------------------------------------------------------------------------------------------------------

-> gender = 1

Logistic regression Number of obs = 5,772

LR chi2(5) = 395.89

Prob > chi2 = 0.0000

Log likelihood = -2796.0498 Pseudo R2 = 0.0661

----------------------------------------------------------------------------------

AKIvar_senest | Odds Ratio Std. Err. z P>|z| [95% Conf. Interval]

-----------------+----------------------------------------------------------------

groupeGFR |

eGFR < 30 | 2.598995 .4196615 5.92 0.000 1.893919 3.56656

30 <= eGFR < 45 | 1.650901 .2324981 3.56 0.000 1.252695 2.175688

45 <= eGFR < 60 | 1.154935 .1463961 1.14 0.256 .9008692 1.480653

60 <= eGFR < 90 | .9216162 .0891081 -0.84 0.399 .7625181 1.11391

|

age50 | 1.025542 .0023843 10.85 0.000 1.020879 1.030225

_cons | .1948169 .0115088 -27.69 0.000 .1735169 .2187316

----------------------------------------------------------------------------------

## **Fully adjusted**

bysort gender: logistic AKIvar_senest ib90.groupeGFR c.age50 diabetes MUT hypertension HF

----------------------------------------------------------------------------------------------------------------------

-> gender = 0

Logistic regression Number of obs = 2,988

LR chi2(9) = 141.08

Prob > chi2 = 0.0000

Log likelihood = -1736.2257 Pseudo R2 = 0.0390

----------------------------------------------------------------------------------

AKIvar_senest | Odds Ratio Std. Err. z P>|z| [95% Conf. Interval]

-----------------+----------------------------------------------------------------

groupeGFR |

eGFR < 30 | 2.152579 .3974211 4.15 0.000 1.499014 3.091098

30 <= eGFR < 45 | 2.31723 .3829741 5.08 0.000 1.676058 3.203681

45 <= eGFR < 60 | 1.768633 .2740482 3.68 0.000 1.305404 2.396242

60 <= eGFR < 90 | 1.002725 .1253121 0.02 0.983 .7848843 1.281025

|

age50 | 1.008743 .0037434 2.35 0.019 1.001432 1.016106

diabetes | 1.416058 .1356538 3.63 0.000 1.17365 1.708533

MUT | .6902996 .2067598 -1.24 0.216 .3837816 1.241627

hypertension | 1.099481 .0968946 1.08 0.282 .9250674 1.306779

HF | 1.172714 .1469998 1.27 0.204 .9172631 1.499306

_cons | .2343736 .0232013 -14.66 0.000 .1930394 .2845584

----------------------------------------------------------------------------------

----------------------------------------------------------------------------------------------------------------------

-> gender = 1

Logistic regression Number of obs = 5,772

LR chi2(9) = 442.00

Prob > chi2 = 0.0000

Log likelihood = -2772.9933 Pseudo R2 = 0.0738

----------------------------------------------------------------------------------

AKIvar_senest | Odds Ratio Std. Err. z P>|z| [95% Conf. Interval]

-----------------+----------------------------------------------------------------

groupeGFR |

eGFR < 30 | 2.323197 .3810924 5.14 0.000 1.684444 3.204168

30 <= eGFR < 45 | 1.486401 .2124796 2.77 0.006 1.123201 1.967045

45 <= eGFR < 60 | 1.084631 .1389742 0.63 0.526 .8437565 1.39427

60 <= eGFR < 90 | .9187631 .0893928 -0.87 0.384 .7592491 1.11179

|

age50 | 1.023036 .0024759 9.41 0.000 1.018195 1.0279

diabetes | 1.548693 .1273296 5.32 0.000 1.318201 1.819487

MUT | 1.197413 .3802143 0.57 0.570 .642632 2.231135

hypertension | 1.205807 .0928341 2.43 0.015 1.036919 1.402204

HF | 1.243305 .1561824 1.73 0.083 .971965 1.590393

_cons | .1720103 .0108051 -28.02 0.000 .1520845 .1945468

----------------------------------------------------------------------------------

## **Test for interaction**

lrtest interaction

Likelihood-ratio test LR chi2(4) = 8.61

(Assumption: interaction nested in .) Prob > chi2 = 0.0716

**Regression analysis including obstructive nephropathy as a confounder**

logistic AKIvar_senest ib90.groupeGFR gender c.age50 diabetes MUT hypertension HF DN13

Logistic regression Number of obs = 8,760

LR chi2(11) = 678.92

Prob > chi2 = 0.0000

Log likelihood = -4494.4467 Pseudo R2 = 0.0702

----------------------------------------------------------------------------------

AKIvar_senest | Odds Ratio Std. Err. z P>|z| [95% Conf. Interval]

-----------------+----------------------------------------------------------------

groupeGFR |

eGFR < 30 | 1.945549 .2408375 5.38 0.000 1.526416 2.479771

30 <= eGFR < 45 | 1.615388 .1758561 4.41 0.000 1.305006 1.999592

45 <= eGFR < 60 | 1.227027 .1215058 2.07 0.039 1.010565 1.489855

60 <= eGFR < 90 | .9263953 .0714544 -0.99 0.322 .7964194 1.077583

|

gender | .8908547 .0490248 -2.10 0.036 .7997685 .9923148

age50 | 1.019745 .002038 9.78 0.000 1.015759 1.023748

diabetes | 1.52041 .0951882 6.69 0.000 1.344837 1.718905

MUT | .8823846 .1940433 -0.57 0.569 .5734189 1.357825

hypertension | 1.180136 .0685592 2.85 0.004 1.05313 1.322459

HF | 1.250397 .1111132 2.51 0.012 1.050529 1.48829

DN13 | 1.836364 .1514177 7.37 0.000 1.56233 2.158463

_cons | .1895572 .0125171 -25.19 0.000 .1665455 .2157486

----------------------------------------------------------------------------------
