# Supplementary material for: Preadmission kidney function and risk of acute kidney injury in patients hospitalized with acute pyelonephritis: A Danish population-based cohort study
Source: PLoS One. 2021 Mar 3;16(3):e0247687. doi: 10.1371/journal.pone.0247687 (PMC7929569; doi:10.1371/journal.pone.0247687)
Supplement: Attachment — Submitted filename: Response to Reviewers.docx [file pone.0247687.s007.docx]

**Point by point response to the editor’s and reviewers’ comments**

**Journal requirements**

**1. Please ensure that your manuscript meets PLOS ONE's style requirements, including those for file naming. The PLOS ONE style templates can be found at**

[**https://journals.plos.org/plosone/s/file?id=wjVg/PLOSOne_formatting_sample_main_body.pdf**](https://journals.plos.org/plosone/s/file?id=wjVg/PLOSOne_formatting_sample_main_body.pdf) **and**

[**https://journals.plos.org/plosone/s/file?id=ba62/PLOSOne_formatting_sample_title_authors_affiliations.pdf**](https://journals.plos.org/plosone/s/file?id=ba62/PLOSOne_formatting_sample_title_authors_affiliations.pdf)

Response: We have filled in the relevant symbol legends (corresponding author and first current address) (l. 4+12 +15-16) and verified that all names are correctly entered.

**2. Please clarify whether there was any ethical oversight over the study.**

Response: According to Danish legislation, this study does not require any ethics permission or statement.

**Was all data used publicly available?**

Response: Please see response to question below.

**3. We note that you have indicated that data from this study are available upon request. PLOS only allows data to be available upon request if there are legal or ethical restrictions on sharing data publicly. For information on unacceptable data access restrictions, please see** [**http://journals.plos.org/plosone/s/data-availability#loc-unacceptable-data-access-restrictions**](http://journals.plos.org/plosone/s/data-availability#loc-unacceptable-data-access-restrictions)**.**

Response: Because of Danish legislation, Danish data cannot be shared publicly. Data are available from the Danish Health Data Authority for researchers who meet the criteria for access to confidential data.

**In your revised cover letter, please address the following prompts:**

**a) If there are ethical or legal restrictions on sharing a de-identified data set, please explain them in detail (e.g., data contain potentially identifying or sensitive patient information) and who has imposed them (e.g., an ethics committee). Please also provide contact information for a data access committee, ethics committee, or other institutional body to which data requests may be sent.**

According to Danish legislation, the authors' own approvals to use the Danish data sources for the current study do not allow them to distribute or make patient data available to other parties. Information on data access is available online (<http://sundhedsdatastyrelsen.dk/da/forskerservice>). Access to data from the Danish Health Data Authority requires approval from the Danish Data Protection Agency (<https://www.datatilsynet.dk/english/the-danish-data-protection-agency/introduction-to-the-danish-data-protection-agency/>). The authors do not have special access privileges to these data.

**b) If there are no restrictions, please upload the minimal anonymized data set necessary to replicate your study findings as either Supporting Information files or to a stable, public repository and provide us with the relevant URLs, DOIs, or accession numbers. Please see** [**http://www.bmj.com/content/340/bmj.c181.long**](http://www.bmj.com/content/340/bmj.c181.long) **for guidelines on how to de-identify and prepare clinical data for publication. For a list of acceptable repositories, please see** [**http://journals.plos.org/plosone/s/data-availability#loc-recommended-repositories**](http://journals.plos.org/plosone/s/data-availability#loc-recommended-repositories)**.**

Response: The above-mentioned prompts are addressed in the revised cover letter. **Additional Editor Comments**

**1.In your paper ,The criteria used for AKI definition as per the guidelines from KDIGO, AKI was defined as an at least 1.5 times relative increase  from preadmission sCr, a relative increase of at least 1.5 in sCr within a period of seven days, or an absolute  increase in sCr of at least 26.5 µmol/l within a period of 48 hours, I was curious how many cases where  diagnosed based on change in creatinine of at least  26.5 µmol/l and what  percentage were diagnosed based on relative increase in creatinine of at least 1.5 from baseline within a period of seven days .As during episode of  AKI creatinine values tend  to fluctuate before it reaches a steady state.**

Response: The Venn diagram below shows how many patients that were diagnosed based on the three different criteria. The majority (n=1,046) were diagnosed based on at least 1.5 times relative increase in preadmission creatinine and this high number could be because the patients already had AKI when they were admitted with pyelonephritis as also suggested by figure 2 showing the 30-day cumulated risk. Most of those did not have any creatinine measurements in the seven days before admission and therefore, they could not fulfill the other two criteria at the day of hospital admission. However, they could potentially fulfill these criteria during follow-up, which is not included in the Venn diagram below.

**2. Most of the time treatment in pyelonephritis varies from 7-14 days depending upon infection underlying condition and organism being treated, just curious to know why the criteria of 30-day risk of acute kidney injury was used. As shorter time frame linked to development of AKI immediately after development of pyelonephritis or to resolution of pyelonephritis makes the correlation easier to understand and clinically significant as anything later after resolution of pyelonephritis may add other factors in development of AKI**

Response: Thank you for this relevant feedback. We agree that the AKI cases found day 15 to 30 may not be directly caused by the pyelonephritis infection, but probably caused by complications, antibiotic treatment or underlying disease. Many of the AKI cases found in the first 14 days after the day of hospital admission could also be caused by complications, antibiotic treatment or underlying disease, as we were unable to distinguish between cases caused by the infection and cases caused by complications, treatment or underlying disease. We argue that including cases occurring on day 15 to 30 is of relevance because their underlying causes may not differ from AKI cases included the first 14 days after hospital admission.

We used the 30-day interval because we were not only interested in the immediate risk of AKI but also AKI as a delayed complication to acute pyelonephritis. Still, to address this issue, we made the sensitivity analysis of AKI within seven days after the day of hospital admission, which showed that 89.3% of the AKI cases happened within one week after hospital admission and yielded similar ORs compared with the primary analysis.

We have now elaborated further on this in the discussion section (l. 297-300):

“Most AKI cases (89.3%) within the 30 days after hospitalization occurred within the first 7 days, which underscores the immediate risk of AKI after hospitalization with pyelonephritis. Complications to pyelonephritis, antibiotic treatment, or underlying disease may contribute to the AKI cases occurring later during the hospitalization.”

**Reviewers' comments
Reviewer's Responses to Questions**

**Comments to the Author**

**1. Is the manuscript technically sound, and do the data support the conclusions?

The manuscript must describe a technically sound piece of scientific research with data that supports the conclusions. Experiments must have been conducted rigorously, with appropriate controls, replication, and sample sizes. The conclusions must be drawn appropriately based on the data presented.**

**Reviewer #1: Yes**

**Reviewer #2: Yes**

**2. Has the statistical analysis been performed appropriately and rigorously?**

**Reviewer #1: Yes**

**Reviewer #2: Yes**

**3. Have the authors made all data underlying the findings in their manuscript fully available?

The** [**PLOS Data policy**](http://www.plosone.org/static/policies.action#sharing) **requires authors to make all data underlying the findings described in their manuscript fully available without restriction, with rare exception (please refer to the Data Availability Statement in the manuscript PDF file). The data should be provided as part of the manuscript or its supporting information, or deposited to a public repository. For example, in addition to summary statistics, the data points behind means, medians and variance measures should be available. If there are restrictions on publicly sharing data—e.g. participant privacy or use of data from a third party—those must be specified.**

**Reviewer #1: Yes**

**Reviewer #2: Yes**

**4. Is the manuscript presented in an intelligible fashion and written in standard English?

PLOS ONE does not copyedit accepted manuscripts, so the language in submitted articles must be clear, correct, and unambiguous. Any typographical or grammatical errors should be corrected at revision, so please note any specific errors here.**

**Reviewer #1: Yes**

**Reviewer #2: Yes**

**5. Review Comments to the Author**

**Reviewer #1:**

**This is a database study that looks at the incidence of AKI in patients admitted with pyelonephritis, stratified by pre-existing CKD. I think that on whole the conclusions support the data. I did notice that the female predominance of pyelonephritis increases with increasing GFR and the male:female ratio at the lowest eGFR was almost equal. That suggests that advanced CKD may predispose to pyelonephritis, which is an interesting observation.**

Response: We agree that it would be interesting to study how CKD affect the risk of pyelonephritis. Unfortunately, this is not possible within the current dataset including only patients with pyelonephritis. The higher proportion of men in patients with low eGFR could also be explained by other risk factors such as prostatic hyperplasia.

While we find it outside the scope of this study to examine this further, it could be an interesting hypothesis to examine in a future study.

**I am also interested by the 10% of cases that occurred more than a week after admission. This may be related to antibiotics but probably does not reflect the infection itself. These points should be addressed in the discussion.**

Response: Thank you for raising this important question. We agree that it is an important point and it is now included in the discussion section (please see lines 297-300).

**Reviewer #2:**

****Please note that the authors have acknowledged that Danish law prohibits the sharing of health data, and this can only be accessed by researchers who meet specific criteria to access this confidential data.

I would like to thank the authors for their painstaking endeavor to provide an interesting insight into the association of AKI and acute pyelonephritis. I hope to offer some constructive criticism to enhance the scientific objectives of the authors:

Language and stylistic considerations:

Line 116 - "Measurements seven days before admission were not included to avoid preadmission eGFR being affected of acute illness due to pyelonephritis." - For the sake of clarity, please consider changing to ""Measurements in the seven days preceding admission were not included to avoid preadmission eGFR being affected of acute illness due to pyelonephritis."**

Response: We have revised as suggested. (l. 120-122) **Line 118 - "...assuming that all patients were Caucasian, which is a reasonable assumption to make of the Danish population." - This is probably a potential source of error for approximately 9% of the population that have a non-Western background (2017 statistics - citation below). I do not disagree with the authors pragmatic approach, but perhaps this potential (relatively minor) source of error could be included as a limitation of the study.**
[**http://www.dst.dk/en/Statistik/emner/befolkning-og-valg/indvandrere-og-efterkommere/indvandrere-og-efterkommere**](http://www.dst.dk/en/Statistik/emner/befolkning-og-valg/indvandrere-og-efterkommere/indvandrere-og-efterkommere)

Response: We have included this as a limitation of the study in the discussion section *(“We also lacked information on ethnicity, but since the majority of the Danish population is Caucasian, this was not expected to substantially influence our results.”*). We have now elaborated further on this issue (l. 284-287):

“We also lacked information on ethnicity. Approx. 9% of the population have a non-Western background and therefore we cannot exclude any minor bias when assuming that all patients are Caucasian [46]. However, since the majority of the Danish population is Caucasian, this was not expected to influence our results substantially [11, 34].” **Line 242-243 - "A total of 89.3% of the AKI cases (i.e. 1,882 cases) 243 occurred within one week after hospital admission." - For the sake of clarity, please consider changing to "A total of 89.3% of the AKI cases (i.e. 1,882 cases) 243 occurred in the first week from the day of hospital admission." The latter sentence makes it clear that the AKI episode occurred relatively early after the acute illness was diagnosed. The original sentence might mislead readers into thinking that AKI may have occurred after the admission concluded (ie. after the patient was discharged).**

Response: We agree and it is changed (l. 243-244). Thank you.  **Methodology / results considerations:

1. Obstructive nephropathy at baseline would be a major risk factor for both chronic kidney disease and for the subsequent development of pyelonephritis. Was this factor considered?**

Response: Thank you for pointing this out. We did not consider obstructive nephropathy in our primary analyses. We have now obtained information on any diagnosis of obstructive nephropathy (ICD-10: N13) within 5 years before hospitalization with pyelonephritis. The prevalence of this condition is tabulated by preadmission eGFR in the table below.

| **Characteristics** | **eGFR**  **< 30** | **eGFR**  **30-44** | **eGFR**  **45-59** | **eGFR**  **60-89** | **eGFR**  **≥ 90** | **Total** |
| --- | --- | --- | --- | --- | --- | --- |
| **N** | 452  (100.00) | 734  (100.0) | 1,076  (100.0) | 3,249  (100.0) | 3,249  (100.0) | 8,760  (100.0) |
| **Obstructive nephropathy (N13)** | 100  (22.1) | 134  (18.3) | 156  (14.5) | 245  (7.5) | 156  (4.8) | 791  (9.0) |

According to the table, obstructive nephropathy is associated with lowered eGFR. As obstructive nephropathy could also be a risk factor for AKI, we have made a sensitivity analysis including obstructive nephropathy as a confounder. The results are shown in the table below.

| **Preadmission eGFR** | **Fully adjusted odds ratios (ORs)** | **95% CI** |
| --- | --- | --- |
| **≥90** | 1.00 *(reference)* | - |
| **60-89** | 0.93 | 0.80 ; 1.08 |
| **45-59** | 1.23 | 1.01 ; 1.49 |
| **30-44** | 1.62 | 1.31 ; 2.00 |
| **<30** | 1.95 | 1.53 ; 2.48 |

In this analysis, the association between preadmission kidney function and AKI persisted but with slightly lower ORs compared with the main analysis. We have included this analysis in the manuscript in the methods (l. 185-186), results (l. 248-249), and discussion (l. 301-303):

l. 185-186:

“We used logistic regression model to conduct a final sensitivity analysis including obstructive nephropathy as a covariate.”

l. 248-249:

“The association between preadmission eGFR and AKI attenuated slightly after adjustment for obstructive nephropathy (S6 Table).”

l. 301-303:

“We found slightly lower ORs when adjusting for obstructive nephropathy (S6 Table). This indicates that some of the found association between preadmission eGFR and AKI could be explained by obstructive nephropathy, but the association persists even after adjustment for this covariate.”

**2. Nephrolithiasis, particularly when obstructive, could result in both AKI and pyelonephritis. This would be an important data point to tease out, if possible. Furthermore, even non-obstructive stones can serve as a nidus of infection and result in a higher likelihood of recurrence of infection (and possibly AKI).**

Response: Thank you for raising this great point. We looked more carefully into this association by obtaining data on any diagnosis of kidney stones (ICD-10: N20 Nephrolithasis) within 5 years before pyelonephritis. We found an almost equal prevalence of kidney stones in the five different exposure groups. Therefore, we argue that it is not an important confounder in the current study.

| **Characteristics** | **eGFR < 30** | **eGFR 30-44** | **eGFR 45-59** | **eGFR 60-89** | **eGFR ≥ 90** | **Total** |
| --- | --- | --- | --- | --- | --- | --- |
| **N** | 452  (100.00) | 734  (100.0) | 1,076  (100.0) | 3,249  (100.0) | 3,249  (100.0) | 8,760  (100.0) |
| **Nephrolithiasis (N20)** | 41  (9.1) | 57  (7.8) | 117  (10.9) | 301  (9.3) | 303  (9.3) | 819  (9.3) |

As a precaution, we have tested the abovementioned assumption in a sensitivity analysis including nephrolithiasis. As expected, the ORs did not differ from the fully adjusted ORs in our main analysis. This latter sensitivity analysis has not been included in the manuscript.

| **Preadmission eGFR** | **Fully adjusted odds ratios (ORs)** | **95% CI** |
| --- | --- | --- |
| **≥90** | 1.00 *(reference)* | - |
| **60-89** | 0.95 | 0.82 ; 1.10 |
| **45-59** | 1.30 | 1.08 ; 1.58 |
| **30-44** | 1.79 | 1.45 ; 2.22 |
| **<30** | 2.19 | 1.72 ; 2.79 |

**In a retrospective analysis, the authors would be limited by the accuracy of ICD-10 data available from these hospitalizations, but I suspect many readers would also want to know if the above points were considered. If this data is not available or too cumbersome to obtain, please consider acknowledging it as potential limitations.**

Response: Please see our responses above. Data on diagnosis codes of obstructive nephropathy (ICD-10: N13) and nephrolithiasis (ICD-10: N20) are available, but since not all patients with these conditions will be admitted to the hospital we do not expect the sensitivity to be complete.  **3. Another interpretation which the authors could consider mentioning is that 89.3% of cases of AKI were diagnosed within the first week from the day of admission, which can likely be attributed to sepsis. However, the remaining ~10% of cases do seem somewhat distant from the original septic insult to be directly attributed to acute pyelonephritis. While the authors do not speculate as to the *cause* of the AKI, it is plausible that potentially nephrotoxic agent exposure (IV contrast, certain antibiotics or NSAIDs for pain relief) may account for the delayed AKI cases. It is hard to say if all this needs to be included, but this could make for some thoughtful discussion, as long as the authors feel that it does not digress too far from their topic of research.**

Response: This is an important point, which was also mentioned by the editor and the other reviewer. Please see our response to the editor comments. We have now included this issue in the discussion section (please see lines 297-300).
